# *Drosophila* Eye Model to Study Neuroprotective Role of CREB Binding Protein (CBP) in Alzheimer’s Disease

**DOI:** 10.1371/journal.pone.0137691

**Published:** 2015-09-14

**Authors:** Timothy Cutler, Ankita Sarkar, Michael Moran, Andrew Steffensmeier, Oorvashi Roy Puli, Greg Mancini, Meghana Tare, Neha Gogia, Amit Singh

**Affiliations:** 1 Premedical Program, University of Dayton, Dayton, Ohio, 45469, United States of America; 2 Department of Biology, University of Dayton, Dayton, Ohio, 45469, United States of America; 3 Center for Tissue Regeneration and Engineering at Dayton (TREND), University of Dayton, Dayton, Ohio, 45469, United States of America; Children's Hospital of Pittsburgh, University of Pittsburgh Medical Center, UNITED STATES

## Abstract

**Background:**

The progressive neurodegenerative disorder Alzheimer’s disease (AD) manifests as loss of cognitive functions, and finally leads to death of the affected individual. AD may result from accumulation of amyloid plaques. These amyloid plaques comprising of amyloid-beta 42 (Aβ42) polypeptides results from the improper cleavage of amyloid precursor protein (APP) in the brain. The Aβ42 plaques have been shown to disrupt the normal cellular processes and thereby trigger abnormal signaling which results in the death of neurons. However, the molecular-genetic mechanism(s) responsible for Aβ42 mediated neurodegeneration is yet to be fully understood.

**Methodology/Principal Findings:**

We have utilized Gal4/UAS system to develop a transgenic fruit fly model for Aβ42 mediated neurodegeneration. Targeted misexpression of human Aβ42 in the differentiating photoreceptor neurons of the developing eye of transgenic fly triggers neurodegeneration. This progressive neurodegenerative phenotype resembles Alzheimer’s like neuropathology. We identified a histone acetylase, CREB Binding Protein (CBP), as a genetic modifier of Aβ42 mediated neurodegeneration. Targeted misexpression of CBP along with Aβ42 in the differentiating retina can significantly rescue neurodegeneration. We found that gain-of-function of CBP rescues Aβ42 mediated neurodegeneration by blocking cell death. Misexpression of Aβ42 affects the targeting of axons from retina to the brain but misexpression of full length CBP along with Aβ42 can restore this defect. The CBP protein has multiple domains and is known to interact with many different proteins. Our structure function analysis using truncated constructs lacking one or more domains of CBP protein, in transgenic flies revealed that Bromo, HAT and polyglutamine (BHQ) domains together are required for the neuroprotective function of CBP. This BHQ domain of CBP has not been attributed to promote survival in any other neurodegenerative disorders.

**Conclusions/Significance:**

We have identified CBP as a genetic modifier of Aβ42 mediated neurodegeneration. Furthermore, we have identified BHQ domain of CBP is responsible for its neuroprotective function. These studies may have significant bearing on our understanding of genetic basis of AD.

## Introduction

Alzheimer’s disease (AD; OMIM: 104300) is an age related progressive neurodegenerative disease. AD is characterized by the loss of neurons in the hippocampus and cortex of the nervous system that results in the loss of cognitive function, memory and finally leading to the death of patients. The most common neurodegenerative dementia AD, which affects ~30 million people worldwide is currently incurable [1, 2]. The causes of AD can be hereditary or acquired. The two characteristic neuropathological hallmarks of AD are: generation of neurofibrillary tangles (NFTs) comprising of hyper-phosphorylated forms of a microtubule associated protein Tau, and accumulation of amyloid beta 42 (Aβ42) plaques [3–11]. The Aβ42 polypeptides are generated by improper cleavage of the Amyloid-Precursor Protein (APP) by β (a membrane bound aspartyl protease also called BACE)- and γ- secretase, which results in a forty-two amino-acid long polypeptide, and referred to as amyloid-beta 42 (Aβ42) [4, 6, 12–15]. These Aβ42 polypeptides form dimers, which results in the partial β structure, assemble into higher orders and results in Aβ aggregates of protein micelles with a hydrophobic core and a polar exterior [13, 15]. Accumulation of Aβ42 plaques are coincident with the abrupt signaling in the neuronal cell [10, 16]. It results in the disruption of major cellular processes and is manifested as oxidative stress, ER stress, generation of reactive oxygen species (ROS), trigger inflammatory processes [8]. It finally results in triggering cell death in the neurons.

It is known that both soluble and insoluble forms of Aβ42 causes aberrant signaling and disrupt cellular processes and thereby lead to AD neuropathology of progressive neurodegeneration [15, 17–19]. Several highly conserved genetic pathways like caspase dependent cell death, Jun-N Terminal kinase (JNK) signaling pathway, apical basal polarity pathway are involved in cell death, cell survival and growth [10, 20–27]. We have seen earlier that these pathways are involved in neuronal cell death due to Aβ42 accumulation. To understand the molecule genetic underpinnings of this complex response due to accumulation of Aβ42 plaques, the animal models like mouse [15, 28] and fruit fly [4–7, 10, 29–33] have been developed and analyzed. However, the exact mechanism is far from fully understood. Nearly 75% of human genes associated with disease have a *Drosophila* ortholog, thus makes *Drosophila* an ideal model to study human disease [34, 35].

The compound eye of fruit fly, *Drosophila melanogaster* can serve as an excellent model to study patterning, growth and survival [3, 8, 10, 34–37]. The compound eye of *Drosophila* develops from an imaginal primordium housed inside the larvae, and is called as eye- antennal imaginal disc. The *Drosophila* eye is an epithelial bi-layer structure which has the blue print for the adult eye and head structures [37–40]. The eye-antennal imaginal disc grows and differentiate into various cell types of the eye. During late second instar of larval development, a synchronous wave of differentiation initiated at the posterior margin of the eye imaginal disc, results in differentiation of retinal neurons or photoreceptor cells from the retinal precursor cells [41]. During larval to pupal metamorphosis, eye imaginal disc differentiates into the pupal retina and later into the adult eye comprising of 800 unit eyes or ommatidia. Each ommatidium comprise of eight photoreceptor cells and pigment and support cells [41–43]. The amenability of Gal4/UAS targeted system allows misexpression of foreign genes along the spatiotemporal axes in the developing eye [10, 44]. We have established a model system in *Drosophila* eye where high levels of human amyloid-beta (Aβ42) are misexpressed in the differentiating retinal neurons of the developing fly retina [10, 24, 26] using a Glass Multiple Repeat Gal4 driver [45]. The GMR-Gal4 driven Aβ42 (GMR-Gal4> UAS-Aβ42, hereafter abbreviated as GMR>Aβ42) transgenic flies exhibited strong neurodegeneration in the fly retina and resulted in highly reduced eye. The penetrance of this GMR>Aβ42 phenotype was 100%, which makes this *Drosophila* eye model a highly reliable tool for identifying the genetic modifiers of the GMR>Aβ42 mediated neurodegeneration [10, 24, 26].

Using these transgenic flies, we misexpressed individually a gene of interest (candidate gene approach) in GMR>Aβ42 background and screened for the rescue of the neurodegenerative phenotype in a simple F1 screen strategy [24]. In another similar screen, we identified CREB Binding Protein (CBP) as one of the genetic modifiers of GMR>Aβ42 mediated neurodegeneration. During development cells destined to form the distinct cell fates receive dynamic combinations of instructions from signaling cascades and are regulated by multifaceted transcriptional complexes comprising of transcriptional coactivators and corepressors. CBP, a member of the CBP/p300 family of proteins [46–48], was first identified by a physical interaction with a CREB transcription factor [49–51]. CBP is a large protein containing more than 3200 amino acids and has several different functional domains [48]. The N-terminus of CBP contains several protein interaction domains including a region that binds hormone receptors and a domain KIX that binds to the CREB as well as other transcription factors [49, 50]. The C-terminal region has (i) Bromo (B) domain that binds to acetylated lysine residue, (ii) a HAT (H) domain that acetylates lysine 8 of histone H4 and (iii) a glutamine (Q) rich stretch that is involved in transcriptional activation [48, 52, 53]. CBP contains several zinc finger motifs for direct DNA binding or protein-protein interactions [54, 55], and physically interacts with more than hundred proteins including acetylated DNA binding proteins (histones during chromatin remodeling), nuclear hormone receptors and terminal member of several signaling transduction cascades [56, 57]. The ability of CBP to simultaneously bind so many diverse factors may be due to its role as a scaffolding protein that link signaling cascades to transcriptional machinery and thereby influence developmental decisions [48, 56, 58, 59]. CBP has potential to influence development through a variety of molecular and biochemical means [48, 60]. CBP functions in neuronal plasticity and cognition [61], in promoting dendritic and axonal morphogenesis, synapse formation and the release of transmitters at neuromuscular junctions [62, 63], head and trunk segments in *Drosophila* early patterning [64–67], and acts as a coactivator of retinal development [47, 68, 69]. Furthermore, CBP mutations in mice, *Drosophila* or human patients exhibit wide range of CBP role in early development and manifests as Rubinstein-Taybi Syndrome [68, 69]. Thus, CBP activity is modular and it plays major role during cell fate specification and survival.

Here we present the role of CBP in Alzheimer’s disease. CBP promotes cell survival by blocking Aβ42 mediated neurodegeneration in the retinal neurons of the *Drosophila* eye. We demonstrate that upregulation of wild-type CBP can significantly rescue Aβ42 mediated neurodegeneration. In addition, CBP levels are reduced in developing retinal cells where Aβ42 levels are increased. Our structure function studies showed that BHQ domains of CBP are required for its neuroprotective function in the *Drosophila* eye.

## Materials and Methods

### Fly Stocks

All the fly stocks used in this study are listed in FlyBase (http://flybase.bio.indiana.edu). The *Drosophila* stocks used in this study are UAS-CBP (FL), a transgene encoding full length wild-type version of CBP protein, and truncated transgenic constructs encoding various domains of CBP protein. We used UAS-CBP^∆Q^ (lacking the C-terminal poly-glutamine rich region), UAS-CBP^∆HQ^ (lacking the HAT domain and the polyglutamine rich domain), UAS-CBP^∆BHQ^ (lacking the bromo domain, the HAT domain and the polyglutamine rich domain), UAS-CBP^∆NZK^ (lacking the N-terminal half comprising of NHR, Zn and Kix domain of CBP protein), UAS-CBP^∆Kix^ (lacking the Kix domain), and UAS-CBP^∆N^ (lacking the N-terminal domain) [47].

We employed the Gal4/Upstream Activator Sequence (Gal4/UAS) system to misexpress the genes of interest in the developing eye [44]. In this study, we used the Glass Multiple Repeat (GMR) driver, which allows misexpression of the transgenes in the differentiating retinal precursor cells of the developing eye imaginal disc as well as the pupal retina and adult eye [45]. We have shown that the misexpression of human Aß42 in the retina (GMR Gal4 > UAS Aß42) shows a stronger phenotype when maintained at 29°C with no penetrance [10]. All Gal4/UAS stocks and crosses were maintained at 18°C, 25°C and 29°C, unless specified, to sample different induction levels.

### Immunohistochemistry

Eye-antennal imaginal discs from wandering third instar larvae were dissected and stained with antibodies following standard protocol [70]. The eye-antennal discs were dissected in 1xPBS, fixed in 4% paraformaldehyde, washed in 1xPBS, then stained with the primary antibodies Rat anti-Elav (1:100), Rat anti-Chaoptin [24B10 (1:100)], Rabbit anti-Dlg (1:200), mouse anti-22C10 (1:100) [71] and Mouse anti-crumbs (1:10). The secondary antibodies used were goat anti-Rat IgG conjugated with Cy5 (1:250), Donkey anti-Mouse IgG conjugated with Cy3 (1:250), and Donkey anti-Mouse IgG conjugated with Cy3. An alternative protocol was used for Crumbs (Crb) staining [26, 72]. After staining, the tissue were mounted in Vectashield (Vector Labs), and all immunofluorescence images were captured using the Olympus Fluoview 1000 Confocal Microscope. The final images and figures were prepared using Adobe Photoshop CS6 software.

### Detection of Cell Death

TUNEL assay was used to detect cell undergoing cell death [73, 74]. TUNEL assays marks the cleaved double and single stranded DNA of dying cells by adding a fluorescently labeled nucleotides to 3’ OH ends in a template-independent manner by Terminal Deoxynucleotidyl Transferase (TdT). It can be detected by fluorescence or confocal microscopy. Eye-antennal discs after secondary antibody staining [75] were blocked in 10% normal donkey serum in phosphate buffered saline with 0.2% Triton X-100 (PBT) and labeled for TUNEL assays using a cell death detection kit from Roche Diagnostics.

The TUNEL positive cells were counted from five sets of imaginal discs and were used for statistical analysis using Microsoft Excel 2010. The P-values were calculated using two-tailed *t*-test and the error bars represent Standard Deviation from Mean [10, 26].

### Adult Eye Imaging

Adult eye phenotypes were screened. Images of the dead flies were taken after they were mounted onto a needle and placed below the lens of the Axioimager.Z1 Zeiss Apotome in a horizontal orientation. The Z-stack feature of the Axiovision software allowed us to capture a stack of images, then later form a single, clear image.

## Results

### CBP is a genetic modifier of Aβ42 mediated neurodegeneration in *Drosophila* eye

**Fig 1:**
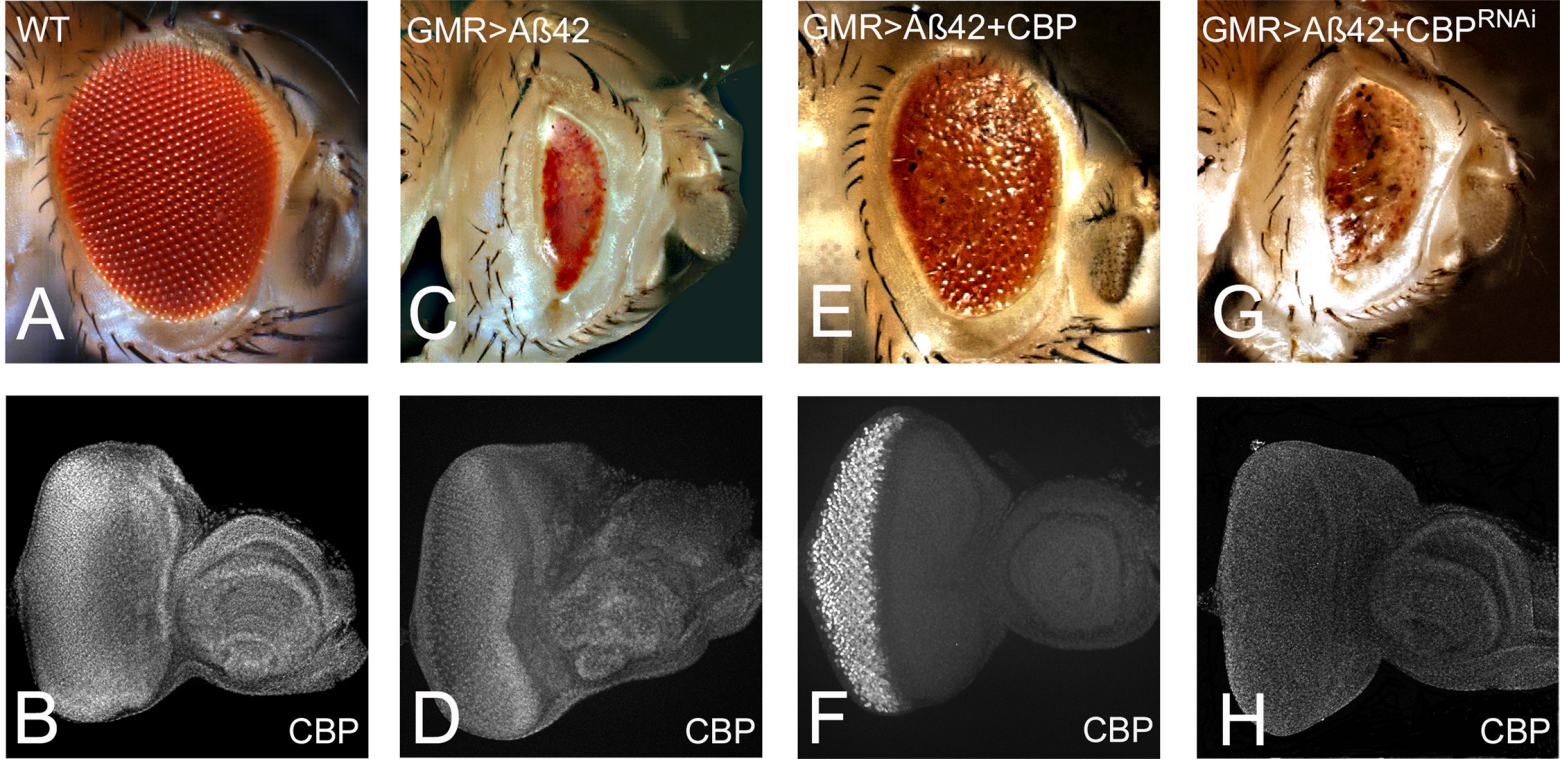
CBP is a genetic modifier of Aβ42 mediated neurodegeneration in the *Drosophila* eye. (A) In comparison to the wild-type compound eye comprising of nearly 800 ommatidia of unit eyes, (C) misexpression of Aβ42 in the differentiating photoreceptor neurons of the developing eye using GMR-Gal4 driver (GMR> Aβ42) results in a strong neurodegenerative phenotype of highly reduced eye, glazed appearance and ommatidial fusion. (E, F) Misexpression of full length CBP with Aβ42 in the developing eye (GMR> Aβ42 +CBP FL) results in a significant rescue of the neurodegeneration in ~20% (22/112) flies as seen in (C) GMR> Aβ42 alone. (G) Reducing CBP function by misexpression of CBP ^RNAi^ (GMR> Aβ42 +CBP ^RNAi^) results in a strong neurodegenerative phenotype in nearly all the hatched flies. Levels of CBP in (B) wild-type, (D) GMR> Aβ42, (F) GMR> Aβ42 +CBP FL, (H) GMR> Aβ42+ CBP ^RNAi^ eye imaginal disc. Note that CBP levels are reduced in GMR> Aβ42 background as compared to the wild-type eye imaginal disc. The orientation of all imaginal discs is identical with posterior to the left and dorsal up. Magnification of all eye imaginal discs is 20X, and adult eyes is 10X.

We have earlier shown that accumulation of Aβ42 triggers cell death of neurons in the *Drosophila* eye [10, 24, 26]. In a forward genetic screen using our *Drosophila* eye model for AD, we have identified a histone acetylase CBP, as a genetic modifier of Aβ42 mediated neurodegeneration. In comparison to the wild type compound eye (Fig 1A), misexpression of Aβ42 in the differentiating neurons of the developing *Drosophila* eye using GMR-Gal4 driver (GMR> Aβ42) resulted in a strong neurodegenerative phenotype of highly reduced adult eye. The GMR> Aβ42 adult eye have glazed appearance due to fusion of lens in 100% flies (Fig 1C, [10]. Misexpression of full length CBP along with Aβ42 in the developing eye (GMR> Aβ42+CBP FL) resulted in a strong rescue of the Aβ42 mediated neurodegeneration in (22/112) ~20% flies (Fig 1E). For each cross more than 100 flies were counted to determine the percentage rescue. We tested the levels of CBP in these background using immuno-histochemical approach and found that CBP which is expressed ubiquitously in the entire eye imaginal disc (Fig 1B) showed significant reduction in the levels in GMR> Aβ42 background (Fig 1D), whereas higher levels of CBP in (GMR> Aβ42+CBP FL) background were coincident with the strong rescue observed (Fig 1E). In order to test that levels of CBP are crucial, we also tested levels of CBP in GMR> Aβ42+CBP^RNAi^ background, where there is no rescue of GMR> Aβ42 mediated neurodegeneration (Fig 1G), and found that CBP levels are reduced and are accompanied with neurodegeneration (Fig 1H). Our data suggest that higher CBP levels can rescue Aβ42 mediated neurodegeneration. Next we wanted to identify the mechanism by which CBP rescued the Aβ42 mediated neurodegeneration.

### Misexpression of CBP can block induction of cell death

**Fig 2:**
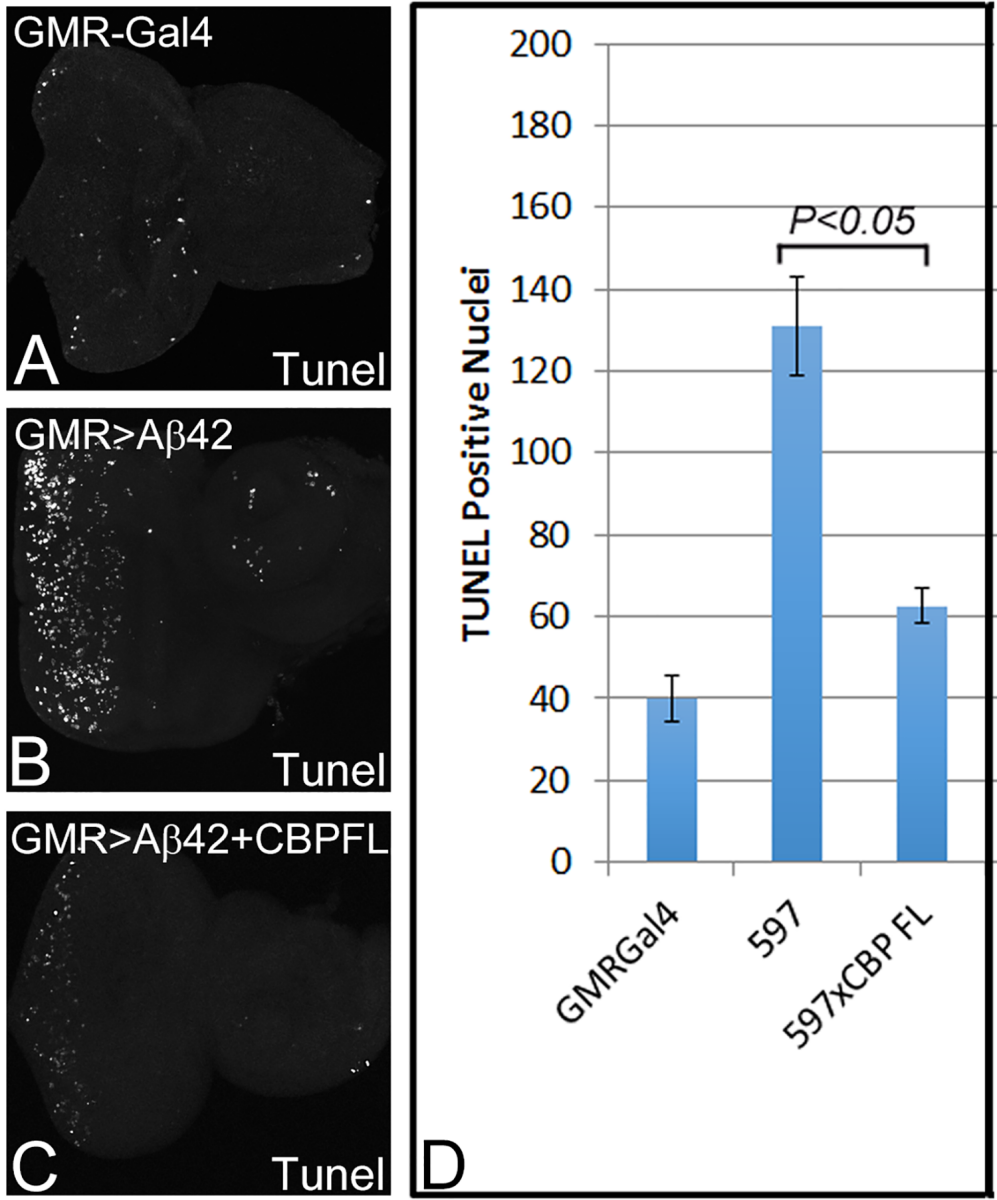
Misexpression of CBP full length can rescue Aβ42 mediated neurodegeneration by blocking cell death. TUNEL staining was performed in third instar eye imaginal disc to mark the dying cells. (A) Wild-type third instar eye imaginal disc showing random cell death in a few cells. (B) The number of dying cells increased significantly in GMR>Aβ42 background, whereas (C) reduced dramatically when full length CBP is misexpressed along with Aβ42 (GMR> Aβ42 +CBP FL) (P = 2.23837E-06). (D) Graph comparing the number of dying cells in the above three backgrounds. The number of TUNEL cells have been recorded and counted from five imaginal disc from all three backgrounds. These rescue phenotype based on number of TUNEL positive cells counted are significant as evident from the calculation of P-values based on the two-tailed t-test using Microsoft Excel 2013. The orientation of all imaginal discs is identical with posterior to the left and dorsal up. Magnification of all eye imaginal discs is 20X.

To test our hypothesis about the role of CBP in Aβ42 mediated neurodegeneration in the *Drosophila* eye, we tested the survival of the retinal cells upon targeted misexpression of both Aβ42 and CBP (GMR> Aβ42+CBP FL) and compared it with the wild-type and GMR> Aβ42 eye imaginal disc (Fig 2). We employed the commonly used TUNEL staining, which marks the fragmented DNA of the dying cells nuclei [73], to discern the mechanism. In the wild-type eye imaginal disc, a few cells are always undergoing cell death as evident from a few random TUNEL positive cells (Fig 2A and 2D). Misexpression of Aβ42 in GMR-Gal4 (GMR> Aβ42) domain resulted in strong induction of cell death as evident from numbers of TUNEL positive cells (Fig 2B and 2D). The increased number of dying cells in GMR> Aβ42 background as compared to the wild-type eye imaginal disc explained the highly reduced adult eye observed in the GMR> Aβ42 background (Fig 1C). The GMR> Aβ42+CBP FL exhibited strong reduction in number of TUNEL positive nuclei (Fig 2C), as compared to the GMR> Aβ42 eye imaginal disc (Fig 2B). In order to quantitate the amount of cell death, we counted the number of dying nuclei in GMR domain in the five eye imaginal disc of all backgrounds. We employed Microsoft Excel 2013 for statistical analysis. The P- values were calculated using one-tailed t-test. The error bars represented the Standard deviation from the Mean [24, 26, 40]. We found that cell death in GMR> Aβ42 background increased more than three-fold from the wild-type imaginal disc (Fig 1D). In GMR> Aβ42+CBP FL background the rate of cell death is significantly reduced as compared to the GMR> Aβ42 background further validating our earlier observation where GMR> Aβ42+CBP FL results in the rescue of GMR> Aβ42 mediated neurodegeneration in the adult eye (Fig 1).

### CBP can restore axonal targeting defects of Aβ42 mediated neurodegeneration

**Fig 3:**
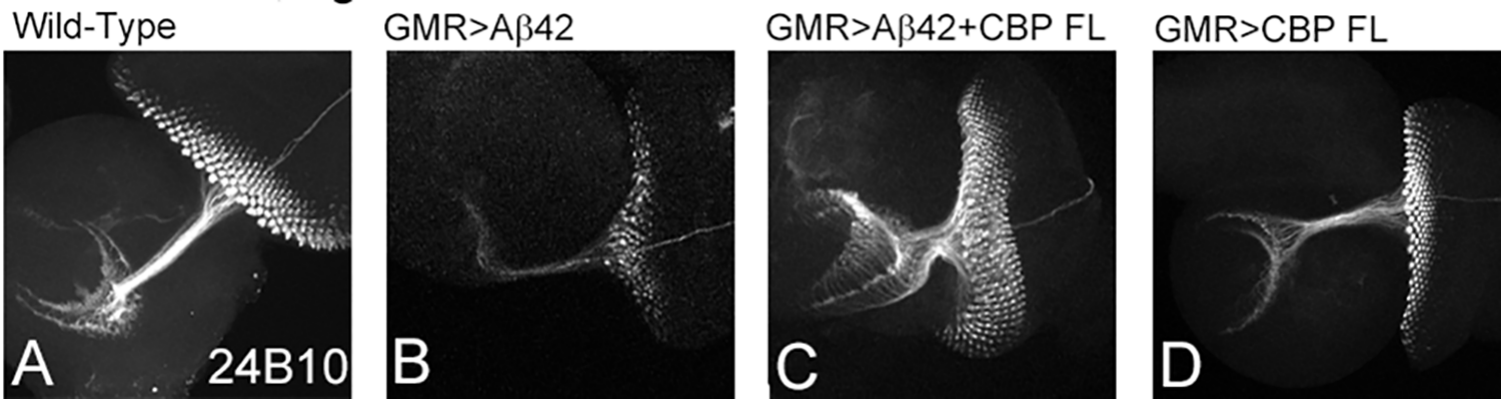
Misexpression of full length CBP can restore axonal targeting impaired by Aβ42 mediated neurodegeneration. The axonal targeting from the retina to the optic lobes of the brain marked by Chaoptin (MAb24B10). (A) In the wild-type eye imaginal disc, the retinal axons marked by MAb24B10 innervates the lamina and medulla of the brain. (B) Misexpression of Aβ42 (GMR> Aβ42) results in impaired axonal targeting whereas (C) misexpression of full length CBP (GMR> Aβ42 +CBP FL) significantly restores the axonal targeting to near wild-type. (D) Note that in control where misexpression of CBP full length alone (GMR> CBP FL) does not impact axonal targeting. The orientation of all imaginal discs is identical with posterior to the left and dorsal up. Magnification of all eye imaginal discs is 20X.

In *Drosophila* eye, the axonal projections from retinal neurons innervate the centers of the brain to generate visual connections [76, 77]. The differentiating retinal neurons in the retian of the developing fly eye form an axonal bundle, which targets the different layers of the brains. Each ommatidium of the *Drosophila* eye comprises of eight photoreceptors (PR1-PR8) where PR1-PR6 innervates lamina and PR7-PR8 extends into medulla of the brain. The Chaoptin (MAb24B10) serves as a reliable marker for retinal axons and their projections to the brain [71]. In the wild-type eye imaginal disc retinal neuron innervate both medulla and lamina in the brain and thus makes a characteristic “inverted cap” like orientation (Fig 3A). However, in the GMR> Aβ42 eye imaginal disc, the retinal axonal targeting gets impaired as evident from highly reduced axonal tract and their innervation points in the brain are also aberrant (Fig 3B). Interestingly, misexpression of CBP FL with Aβ42 (GMR> Aβ42 +CBP FL) significantly restores the axonal targeting aberrations (Fig 3C) to near wild-type (Fig 3A) in nearly 26% (8/30) of the imaginal discs stained and imaged. The controls showing misexpression of full length CBP alone in GMR domain (GMR>CBPFL) shows wild-type axonal targeting (Fig 3D).

### BHQ domain of CBP is required for its neuroprotective function in Aβ42 mediated neurodegeneration

**Fig 4:**
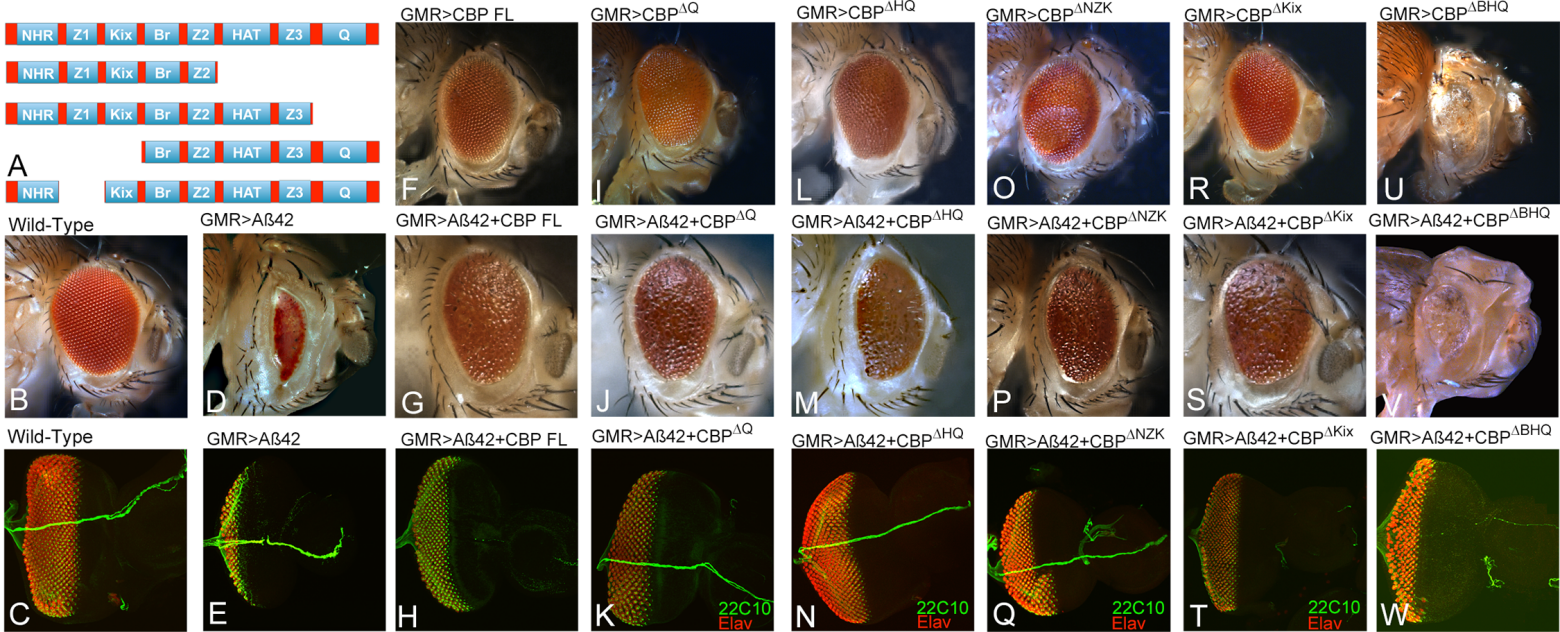
BHQ (Bromo, HAT and poly Glutamine stretch) domain is required for CBP function in rescuing Aβ42 mediated neurodegeneration. (A) In order to functionally dissect the requirement for various domains of the complex CBP protein, various different variants lacking the domains of CBP were used to determine the neuroprotective function of CBP. (B, C) Wild type adult eye and eye imaginal disc stained with Elav (Red) and 22C10 (green). (D, E) GMR> Aβ42 showing strong neurodegenerative phenotype as seen in the (D) adult eye and (E) eye imaginal disc. (F, G, H) misexpression of (F) CBP full length alone (GMR>CBP FL) serve as control and exhibits a normal eye, whereas (G, H) misexpression of CBP full length with Aβ42 (GMR> Aβ42 +CBP FL) results in a significant rescue as seen in the (G) adult eye (22/112) and (H) eye imaginal disc. (I, J, K) Misexpression of CBP^∆Q^ (I) alone (GMR>CBP^∆Q^) serve as control and exhibits a normal eye, whereas (J, K) misexpression of CBP ^∆Q^ with Aβ42 (GMR> Aβ42 +CBP^∆Q^) results in a significant rescue as seen in the (J) adult eye (23/109) and (K) eye imaginal disc. (L, M, N) Misexpression of CBP^∆HQ^ (L) alone (GMR>CBP^∆HQ^) serve as control and exhibits a normal eye, whereas (M, N) misexpression of CBP^∆HQ^ with Aβ42 (GMR> Aβ42 +CBP^∆HQ^) results in a significant rescue as seen in the (M) adult eye (29/137) and (N) eye imaginal disc. (O, P, Q) Misexpression of CBP^∆NZK^ (I) alone (GMR>CBP^∆NZK^) serve as control and exhibits a normal eye, whereas (P, Q) misexpression of CBP ∆NZK with Aβ42 (GMR> Aβ42 +CBP^∆NZK^) results in a significant rescue as seen in the (J) adult eye (26/119) and (K) eye imaginal disc. (R, S, T) Misexpression of CBP^∆Kix^ (I) alone (GMR>CBP^∆Kix^) serve as control and exhibits a normal eye, whereas (S, T) misexpression of CBP^∆Kix^ with Aβ42 (GMR> Aβ42 +CBP^∆Kix^) results in a significant rescue as seen in the (S) adult eye (21/103) and (T) eye imaginal disc. (U, V, W) Misexpression of CBP^∆BHQ^ (I) alone (GMR>CBP ^∆BHQ^) exhibits reduced eye phenotype, whereas (S, T) misexpression of CBP^∆BHQ^ with Aβ42 (GMR> Aβ42 +CBP^∆BHQ^) results in a dramatic enhancement of neurodegenerative phenotype as seen in the GMR> Aβ42 (V) adult eye (11/11 unhatched pupae) and (W) eye imaginal disc. The P- values calculated for the phenotypes counted for all these constructs were significant. Magnification of all eye imaginal discs is 20X, and adult eyes is 10X.

CBP encodes a complex protein which has multiple interaction domains. We tested several CBP variants lacking either single or multiple domains (Fig 4A) [47]. We employed these constructs in our targeted misexpression approach to discern which domain is required for the CBP mediated rescue of neurodegeneration. In comparison to the wild type eye imaginal disc and adult eye (Fig 4B and 4C), misexpression of Aβ42 results in a strong neurodegeneration as seen in the adult eye (Fig 4D) and to some extent in eye imaginal disc (earlier stages of eye development, Fig 4E). Misexpression of full length CBP alone (GMR> CBP FL) does not show any eye phenotypes (Fig 4F) whereas along with Aβ42 in the differentiating retinal cells (GMR> Aβ42 +CBP FL), it results in significant rescue of neurodegeneration (Fig 4G and 4H). Misexpression of truncated CBP transgenes lacking the polyglutamine rich domain alone (GMR> CBP^∆Q^) does not show any eye phenotypes (Fig 4I) whereas along with Aβ42 in the differentiating retinal cells (GMR> Aβ42 +CBP ^∆Q^), it results in significant rescue of neurodegeneration (Fig 4J and 4K). Misexpression of truncated CBP transgenes lacking the HAT and polyglutamine rich domain (GMR> CBP^∆HQ^) does not show any eye phenotypes (Fig 4L) whereas along with Aβ42 in the differentiating retinal cells (GMR> Aβ42 +CBP^∆HQ^), it results in significant rescue of neurodegeneration (Fig 4M and 4N). Misexpression of truncated CBP transgenes lacking the N-terminal half comprising of NHR, Zinc finger and Kix domain of CBP protein (GMR> CBP^∆NZK^, Fig 4O) and Kix domain (GMR> CBP^∆KIX^, Fig 4R) does not show any eye phenotypes whereas along with Aβ42 in the differentiating retinal cells (GMR> Aβ42 +CBP ^∆NZK^, Fig 4P and 4Q), (GMR> Aβ42 +CBP ^∆KIX^, Fig 4S and 4T) it results in significant rescue of neurodegeneration. Thus, removing any of these domains still exhibits rescue as seen with CBP FL. However, misexpression of truncated CBP transgenes lacking the Bromo, HAT and polyglutamine rich domain (GMR> CBP ^∆BHQ^) show weak neurodegenerative eye phenotypes (Fig 4U) and with Aβ42 in the differentiating retinal cells (GMR> Aβ42 +CBP ^∆BHQ^) also results in significant enhancement of neurodegeneration (Fig 4V and 4W). All the flies of GMR> Aβ42 +CBP ^∆BHQ^ failed to hatch out and were dissected out from the pupal case. This data suggests that BHQ domain of CBP is required for the neuroprotective function of CBP.

### BHQ domain of CBP is required to block cell death induced by Aβ42 accumulation

**Fig 5:**
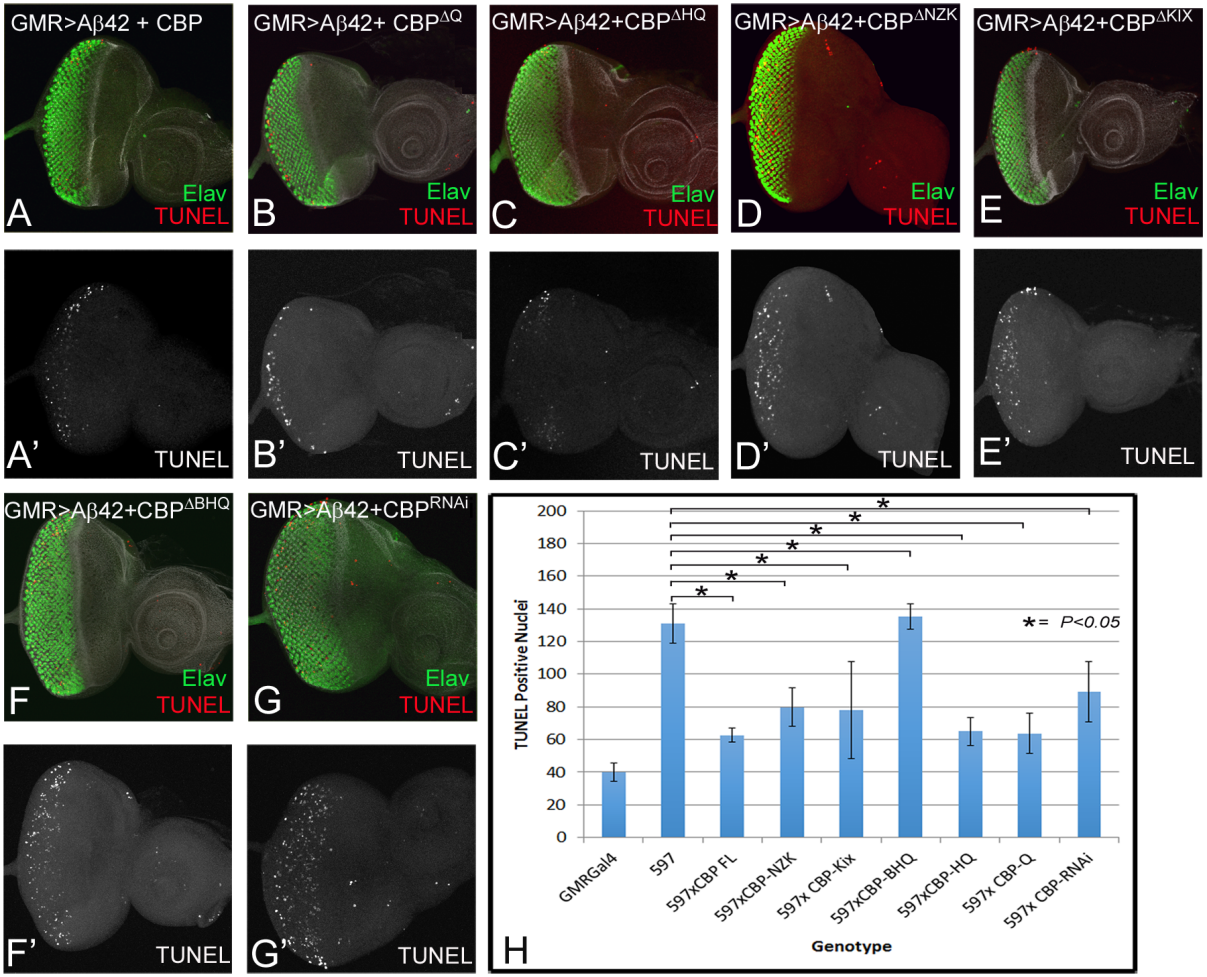
BHQ (Bromo, HAT and poly Glutamine stretch) domain is required for neuroprotective function of CBP. In third instar eye imaginal disc, TUNEL staining was employed to mark the dying cells. In comparison to GMR>Aβ42 background, (A, A’) the number of dying cells marked by TUNEL are significantly reduced when CBP full length is misexpressed along with Aβ42 (GMR> Aβ42 +CBP FL) in the *Drosophila* eye. (B-E) The number of dying cells marked by TUNEL staining in (B, B’) GMR>Aβ42 +CBP^∆Q^, (C, C’) GMR> Aβ42 +CBP^∆HQ^, (D, D’) GMR>CBP^∆NZK^, (E, E’) GMR> Aβ42 +CBP^∆Kix^ background were comparable to (A, A’) GMR> Aβ42 +CBP FL background. However, the number of dying cells marked by TUNEL staining in (F, F’) GMR> Aβ42 +CBP^∆BHQ^ and (G, G’) GMR> Aβ42 +CBP^RNAi^ were significantly increased. (H) Graph comparing the number of dying cells in the all the backgrounds tested. The number of TUNEL cells counted from five imaginal disc of all backgrounds were used for 2-tailed test and P values (< .05). These rescue phenotype based on number of TUNEL positive cells counted are significant. Magnification of all eye imaginal discs is 20X.

To validate our hypothesis, we analyzed and quantitated Aβ42 mediated cell death in various backgrounds expressing various CBP variants (Fig 4A). We wanted to ascertain if neuroprotective function of CBP, which is dependent on BHQ domain, is mediated through by blocking cell death. Misexpression of full length CBP (GMR> Aβ42 +CBP FL) reduces cell death (Fig 5A and 5A’) as compared to the GMR> Aβ42 background (Figs 2B and 5H). Furthermore misexpression of truncated form of CBP in Aβ42 background such as GMR> Aβ42 +CBP ^∆Q^ (Fig 5B and 5B’), GMR> Aβ42 +CBP ^∆HQ^ (Fig 5C and 5C’), GMR> Aβ42 +CBP ^∆NZK^ (Fig 5D and 5D’), GMR> Aβ42 +CBP ^∆KIX^ (Fig 5E, 5E’ and 5H) showed suppression in number of cell dying due to cell death, which was comparable to that seen in the CBP full length expression (Fig 5A and 5H). However, misexpression of GMR> Aβ42 +CBP ^∆BHQ^ resulted in induction of cell death in a number of retinal cells which was comparable to that of GMR> Aβ42 (Figs 2B and 5H). We found similar effects with GMR> Aβ42 +CBP^RNAi^ (Fig 5G, 5G’ and 5H). Thus, more than three-fold increase in cells undergoing cell death due to misexpression of Aβ42 can be reduced to less than two-fold by misexpression of full length CBP whereas the number of dying cells is further increased in CBP variant lacking BHQ domain (Fig 5H), further validating our observation that BHQ domain is required for the neuroprotective function of CBP in Aβ42 mediated neurodegeneration.

### BHQ domain of CBP is required to restore Aβ42 mediated axonal targeting defects

**Fig 6:**
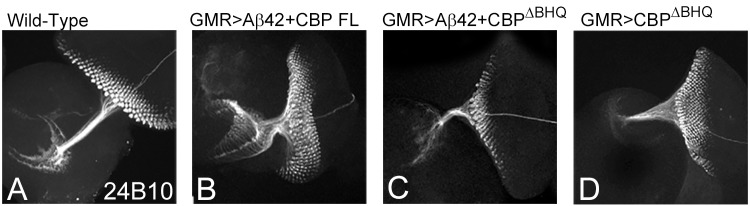
BHQ (Bromo, HAT and poly Glutamine stretch) domain is required for CBP function of restoring axonal targeting. The axonal targeting from the retina to the brain can be studied using the Chaoptin (MAb24B10) marker (REF). (A) In the wild-type eye imaginal disc, the retinal axons marked by MAb24B10 innervates the lamina and medulla of the brain. Misexpression of Aβ42 (GMR> Aβ42) results in impaired axonal targeting. (B) Misexpression of full length CBP (GMR> Aβ42 +CBP FL) as well as (C) CBP variant lacking BHQ domain (GMR> Aβ42 +CBP ^∆BHQ^) significantly restores the axonal targeting to near wild-type. (D) Note that in control where misexpression of CBP variant lacking BHQ domain (GMR>CBP ^∆BHQ^) also affect axonal targeting. Magnification of all eye imaginal discs is 20X.

Tthe *Drosophila* visual system comprises of differentiating retinal neurons sending axonal projections precisely to their synaptic targets in the different layers of the brain [78, 79]. The axons from photoreceptors (PRs) 1–6 terminate in the lamina whereas PR7-PR8 targets the medulla. The retinal axonal connection to the brain can be marked by Chaoptin (MAb24B10) [71]. In the wild-type eye imaginal disc MAb24B10 marks the axons projecting from retinal neurons to the optic lobe of the brain (Fig 6A). However, in the GMR>Aβ42 eye imaginal disc, the retinal axon targeting becomes impaired as the axons are truncated and no longer innervate precisely in the optic lobes of the brain (Fig 3B). Misexpression of *CBP FL* in the GMR>Aβ42 background (GMR>Aβ42+CBP FL) can not only restore the size of the eye field but can also significantly restore the retinal axon targeting from retinal neurons to the two different layers of the brain (Fig 6B). However, misexpression of CBP variant lacking BHQ domain (GMR> Aβ42 +CBP^∆BHQ^) exhibit strong enhancement of mutant phenotype of loss of axonal targeting (Fig 6C). In controls, GMR> CBP^∆BHQ^ also an aberrant axonal targeting was seen (Fig 6D). However, the frequency and extent of phenotype was less severe.

### Misexpression of CBP downregulated *crumbs* (*crb*) levels in Aβ42 background

**Fig 7:**
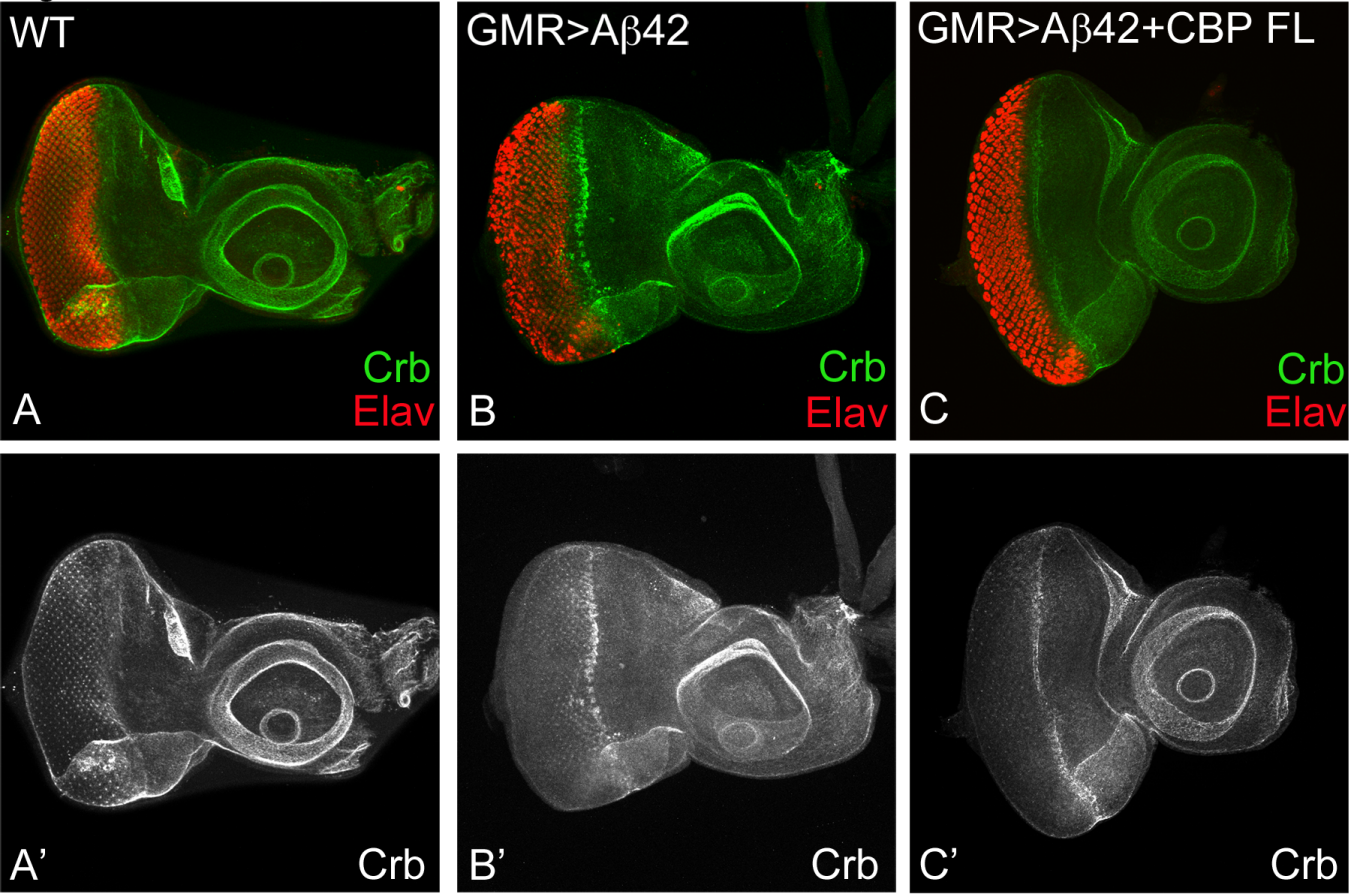
Neuroprotective function of CBP may be mediated through Crb. (A, A’) Wild-type expression of Crb, an apical basal polarity gene, in third instar eye imaginal disc. (B, B) In comparison Crb level are increased in the GMR> Aβ42 background. (C, C’) Crb levels are reduced in GMR> Aβ42+ CBP background. (A’, B’ C’) split channels to show Crb levels. Magnification of all eye imaginal discs is 20X.

An apical basal polarity gene, *crb*, can serve as a marker for Aβ42 mediated neurodegeneration [26]. In comparison to wild-type Crb levels in the developing third instar eye imaginal disc (Fig 7A and 7A’), misexpression of Aβ42 (GMR>Aβ42) resulted in strong induction of Crb levels in the GMR expression domain marking the differentiating retinal precursor cells (Fig 7B and 7B’). Thus, we have shown earlier that Crb expression is increased in Aβ42 background [26]. Misexpression of full length CBP in Aβ42 background (GMR>Aβ42+CBP FL) showed strong rescue of neurodegeneration, which was also accompanied by reduced Crb expression (Fig 7C and 7C’). This strongly suggests that *crb*, which is induced in Aβ42 background is downregulated by misexpression of CBP full length.

## Discussion

The characteristic features of neurodegenerative disorders like Parkinson, Alzheimer’s is an age related manifestation of progressive neurodegeneration. This neurodegeneration is an outcome of impairment of signaling pathways which inhibit basic cellular process and thereby leading to the onset of cell death in the neurons. It is known that these complex neurodegenenerative disorders are not an outcome of a single gene mutation but misregulation of several different pathways. Earlier studies have generated several molecular genetic insights into the death of the neurons. However, our understanding of these complex neurodegenerative disorders is far from complete [4, 11]. In AD, it is known that accumulation of amyloid plaques trigger the neurodegenerative response [4, 13]. It has been shown that basic cell biological processes related to cell death and cell survival are impaired in the Aβ42 mediated neurodegeneration [8, 10].

### BHQ domain of CBP exhibits novel neuroprotective function in AD

Using our *Drosophila* eye model [10, 24, 26], we identified CREB binding protein (CBP) as a genetic modifier of Aβ42 mediated neurodegeneration. Earlier CBP has been shown to modulate poly glutamine (poly Q) disease [80–82]. Our studies tested the domains of CBP protein responsible for its role as a neuroprotective agent in Aβ42 mediated neurodegeneration and found that the N-terminus of this >3200 amino acid long protein [48, 49, 53], which contains several interaction domains including the NHR domain that binds nuclear hormone receptors, Zinc finger domains and KIX domain that binds CREB transcription factor [46, 47] is/are not required for Aβ42 mediated neurodegeneration. The C-terminal half of CBP, which has Bromo domain that binds to acetylated lysine residue, a HAT domain that acetylates lysine 8 of histone H4 and a glutamine rich stretch implicated in transcriptional activation were found to be responsible for Aβ42 mediated neurodegeneration. In addition, we found that C- terminal BHQ domain is required for neuroprotective function whereas the HAT and Poly Q alone or together are not sufficient to demonstrate the neuroprotective function of CBP (Figs 4 and 5). Thus, CBP binding function or nuclear hormone receptor mediated interaction of CBP is not required for the Aβ42 mediated neurodegeneration. Interestingly, CBP, a transcriptional coactivator with intrinsic histone acetyl transferase (HAT) activity depends on the bromodomain of CBP [55]. Our studies demonstrated that HAT, Bromo and Poly Q domain are required for the neuroprotective role of CBP in Aβ42 mediated neurodegeneration.

### Neuroprotective function of CBP is independent of its role in retinal development

Interestingly, CBP is a complex protein which has capability to simultaneously bind so many diverse factors suggest that it may be involved in different cell biological processes along the spatio-temporal axis. It has been shown that CBP full length, CBP^∆HQ^ and CBP^∆BHQ^ domain are sufficient to support photoreceptor development and also to promote expression of eye specification genes in the *Drosophila* eye. Since these studies have been carried out in the *Drosophila* eye, the obvious question was to test whether this function of CBP overlaps with that of its role in photoreceptor development. Our studies show that only BHQ domain is required for neuroprotective function rules out the possibility that the rescue of neurodegeneration in full length CBP and other constructs may not mediated through its eye specification function. Both HQ and BHQ domain has CREB and Kix domain, these domains bind to Cubitus interruptus (Ci), the terminal member of the Hedgehog (Hh) Signaling pathway.

Secondly, in our transgenic model the Aβ42 expression is triggered at the time of retinal differentiation using a GMR Gal4 driver [10, 45], which is much later than the event of eye specification and the onset of retinal determination and differentiation genes expression. Thus, eye specification function of CBP may be independent of its neuroprotective function in Aβ42 mediated neurodegeneration. CBP is known to play roles in many developmental decisions in the developing eye [46, 47].

### CBP neuroprotective function is dependent on Crb levels

Earlier we have demonstrated that an apical-basal polarity gene *crb* plays a role in Aβ42 mediated neurodegeneration. We also found that higher levels of Crb are associated with Aβ42 mediated neurodegeneration [26]. Thus, Crb can serve as an excellent marker for the induction of cell death due to accumulation of Aβ42 in the developing eye [26]. During *Drosophila* wing development, the transcription of apical basal polarity gene *crb* is upregulated by Notch (N) signaling at the dorso-ventral (DV) boundary. In the developing eye, DV boundary organization requires N function. Crb is known to act as negative regulator of N signaling pathway [83]. Furthermore, Crb can inhibit the activity of gamma-secretase to refine N activity domain [84]. Both APP and N are cleaved by similar secretases [85], raises a possibility that Crbs may affects Aβ42 mediated neurodegeneration through N signaling pathway. We have demonstrated earlier Crb levels are upregulated in Aβ42 mediated neureodegeneration. Our data suggests that CBP FL misexpression can promote its neuroprotective function by downregulating Crb levels.

These studies will have significant bearings on understanding the genetic basis of Aβ42 mediated neurodegeneration. It has been seen that individuals with reduced CBP levels have cognitive defects linked to neurological disorders. Earlier it has been argued that CBP may not be a suitable therapeutic target as CBP function is required in many basic cell biological processes. However, recently Naphthol AS-TR phosphate (NASTRp), a novel small molecule inhibitor of CREB-CBP complex has been suggested to possess anti–cancer effects in lung cancers [86]. Furthermore, CBP inhibitors like ICG-001 were shown to suppress pancreatic cancers [87]. Additionally, CBP may mediate the interaction between signaling pathways and these selector genes may be participating in the neuroprotective function of CBP. Thus, further studies focusing on downstream targets might result in identification of possible therapeutic target [88]. Finally, our *Drosophila* eye model [10], can be an excellent choice to test these inhibitors in their role in Aβ42 mediated neurodegeneration.
